# Mutant KRAS regulates transposable element RNA and innate immunity via KRAB zinc-finger genes

**DOI:** 10.1016/j.celrep.2022.111104

**Published:** 2022-07-19

**Authors:** Roman E. Reggiardo, Sreelakshmi Velandi Maroli, Haley Halasz, Mehmet Ozen, Eva Hrabeta-Robinson, Amit Behera, Vikas Peddu, David Carrillo, Erin LaMontagne, Lila Whitehead, Eejung Kim, Shivani Malik, Jason Fernandes, Georgi Marinov, Eric Collisson, Angela Brooks, Utkan Demirci, Daniel H. Kim

**Affiliations:** 1 Department of Biomolecular Engineering, University of California, Santa Cruz, Santa Cruz, CA 95064, USA; 2 Department of Molecular, Cell, and Developmental Biology, University of California, Santa Cruz, Santa Cruz, CA 95064, USA; 3 Canary Center at Stanford for Cancer Early Detection, Department of Radiology, Stanford University School of Medicine, Palo Alto, CA 94305, USA; 4 Broad Institute of MIT and Harvard, Cambridge, MA 02142, USA; 5 Department of Medical Oncology, Dana-Farber Cancer Institute, Boston, MA 02215, USA; 6 Department of Medicine, University of California, San Francisco, San Francisco, CA 94158, USA; 7 Department of Genetics, Stanford University School of Medicine, Stanford, CA 94305, USA; 8 Institute for the Biology of Stem Cells, University of California, Santa Cruz, Santa Cruz, CA 95064, USA; 9 Genomics Institute, University of California, Santa Cruz, Santa Cruz, CA 95064, USA; 10 Center for Molecular Biology of RNA, University of California, Santa Cruz, Santa Cruz, CA 95064, USA; 11 Lead contact

## Abstract

RAS genes are the most frequently mutated oncogenes in cancer, yet the effects of oncogenic RAS signaling on the noncoding transcriptome remain unclear. We analyzed the transcriptomes of human airway and bronchial epithelial cells transformed with mutant KRAS to define the landscape of KRAS-regulated noncoding RNAs. We find that oncogenic KRAS signaling upregulates noncoding transcripts throughout the genome, many of which arise from transposable elements (TEs). These TE RNAs exhibit differential expression, are preferentially released in extracellular vesicles, and are regulated by KRAB zinc-finger (KZNF) genes, which are broadly downregulated in mutant KRAS cells and lung adenocarcinomas *in vivo*. Moreover, mutant KRAS induces an intrinsic IFN-stimulated gene (ISG) signature that is often seen across many different cancers. Our results indicate that mutant KRAS remodels the repetitive noncoding transcriptome, demonstrating the broad scope of intracellular and extracellular RNAs regulated by this oncogenic signaling pathway.

## In brief

Many human cancers are driven by mutant KRAS, but its effects on noncoding RNA are unclear. Reggiardo et al. show that mutant KRAS regulates this RNA landscape by silencing KRAB zinc-finger genes that normally repress transposable element noncoding RNAs, which are preferentially released from mutant KRAS cells in extracellular vesicles.

## Graphical Abstract

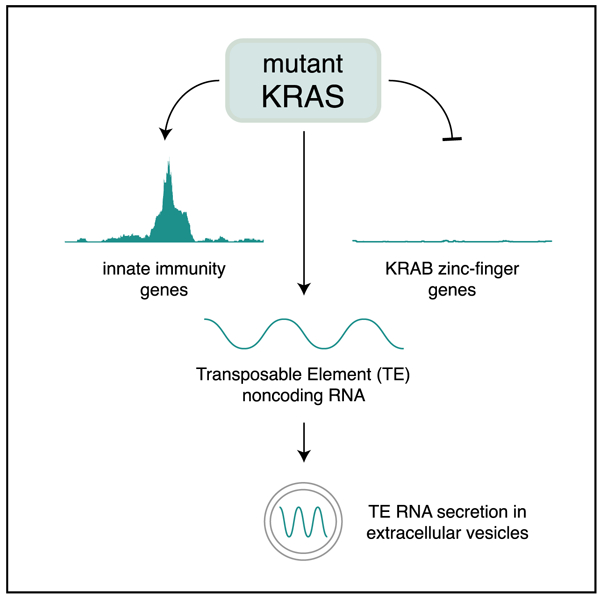

## INTRODUCTION

Most of the human genome is noncoding and transcribed into RNA (Djebali et al., 2012). Moreover, about half of the human genome is comprised of transposable elements (TEs) (Lander et al., 2001), and TEs contribute substantially to the noncoding transcriptome (Kelley and Rinn, 2012; Rinn and Chang, 2020). TE RNAs (Burns, 2017) and other classes of noncoding RNAs are often altered during cancer (Slack and Chinnaiyan, 2019) and epigenetic reprogramming (Kim et al., 2015), where activation of RAS signaling leads to the repression of microRNAs (Kent et al., 2010) and the upregulation of long noncoding RNAs (lncRNAs) (Kim et al., 2015), respectively, via changes in chromatin accessibility. In lung cancers, RAS mutations are present in one-third of lung adenocarcinomas (Cancer Genome Atlas Research, 2014) and serve as driver mutations that initiate tumorigenesis (Jackson et al., 2001). Although RAS genes are among the most frequently mutated oncogenes in cancer (Simanshu et al., 2017), how oncogenic RAS signaling regulates the noncoding transcriptome remains unknown.

To investigate the role of mutant KRAS in reprogramming the transcriptome during early stages of cellular transformation, we characterized the composition of both intracellular and extracellular RNA, including protein-coding RNA, lncRNA, and TE RNA, using human airway epithelial cells (Lundberg et al., 2002) and human bronchial epithelial cells (Ramirez et al., 2004) with constitutively active mutant KRAS. We show that oncogenic KRAS induces TE RNA and cell-intrinsic interferon (IFN)-stimulated gene (ISG) signatures and that KRAB zinc finger (KZNF) genes are globally downregulated both *in vitro* and in mutant KRAS lung adenocarcinomas *in vivo*. Moreover, our findings indicate that significant upregulation and extracellular secretion of TE RNAs and ISGs are transcriptomic signatures of mutant KRAS signaling.

## RESULTS

### Transcriptomic reprogramming by mutant KRAS

**Figure 1.**
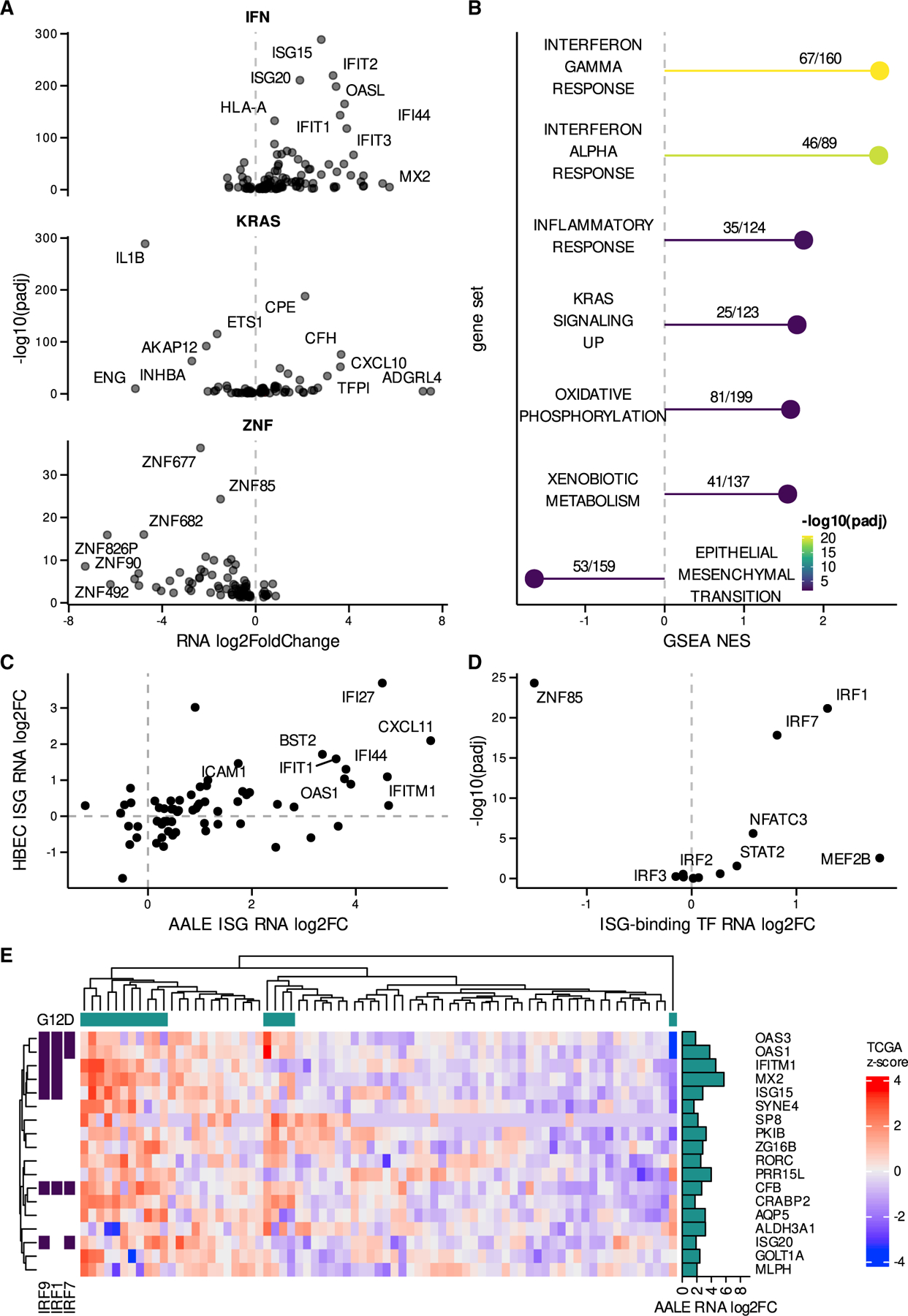
Mutant KRAS signaling activates an intrinsic ISG signature (A) Volcano plots depicting significant differential expression observed in key gene sets (interferon [IFN] response alpha/gamma: IFN, KRAS signaling up: KRAS, zinc-finger genes: ZNF). (B) Significant gene set enrichment analysis (GSEA) results observed in mutant KRAS AALE differentially expressed genes ranked by adjusted p value (padj), normalized enrichment score (NES), and annotated with the number of genes observed out of the total genes in each gene set. (C) Differential expression of ISGs in mutant KRAS AALEs compared to mutant KRAS HBECs. (D) Differentially expressed transcription factors (TFs) with binding motifs enriched in differentially expressed ISG promoter regions. (E) Hierarchical clustering of expression *Z* score in TCGA LUAD RNA-seq data for ISGs upregulated in mutant AALE and exhibiting strong segregation in TCGA LUAD samples based on KRAS G12D mutation status; presence of IRF9/1/7 binding motifs in promoter regions of labeled ISGs.

To determine the transcriptomic landscape of protein-coding and noncoding RNAs regulated by oncogenic RAS signaling, we performed RNA sequencing (RNA-seq) on human airway epithelial cells (AALE) that undergo malignant transformation upon the introduction of mutant KRAS (Lundberg et al., 2002). We compared the transcriptomes of AALE cells transduced with control lentiviral vector to AALEs that were transduced by mutant KRAS-containing lentiviral vector and performed differential expression analysis. We identified thousands of significantly differentially expressed protein-coding RNAs (n = 1,028 upregulated, n = 1,194 downregulated), including ISGs, KRAS signaling genes, and zinc-finger genes (Figures 1A and S1A), as well as hundreds of significantly differentially expressed lncRNAs (n = 116 upregulated, n = 163 downregulated) (Figure S1A), demonstrating the broad extent to which mutant KRAS reprograms the transcriptome (Figures S1A and S1B).

### Mutant KRAS induces intrinsic ISG expression

To explore the biological pathways that are perturbed by oncogenic RAS signaling, we performed gene set enrichment analysis (GSEA) (Powers et al., 2018) using genes that were differentially expressed in our mutant KRAS AALE cells. GSEA revealed that the three most significantly enriched pathways were the IFN-*α* and -γ responses, as well as the hallmark inflammatory response (Figure 1B), along with increased KRAS signaling from mutant KRAS(G12D), increased metabolic gene expression, and decreased expression of epithelial-to-mesenchymal transition (EMT) genes (Figure 1B).

To further validate the connection observed between mutant KRAS and ISG expression, we compared mutant KRAS-induced ISGs in AALE cells to those that were induced in human bronchial epithelial cells (HBECs) in response to mutant KRAS(G12V) (Figure S1C). We observed a strong concordance between mutant KRAS-induced ISGs in AALE and HBEC cells (Figure 1C), confirming our previous results. We then examined the promoter regions (±500 bp) of upregulated ISGs and identified motifs enriched in comparison to non-differentially expressed (DE) ISGs (Figure 1D), including the key IFN response regulators IRF1, IRF7, and STAT2 (Jefferies, 2019). To determine the *in vivo* relevance of our findings in both mutant KRAS AALE and HBEC cells, we examined ISG expression in mutant KRAS(G12D) lung adenocarcinomas (LUAD) from The Cancer Genome Atlas (TCGA), which revealed a subset of ISGs that were upregulated in KRAS(G12D) tumors when compared to lung cancer samples with wild-type (WT) KRAS (Figure 1E). These results indicate that mutant KRAS signaling activates an intrinsic ISG response in lung cells both *in vitro* (AALE, HBEC) and *in vivo* (TCGA LUAD).

### Epigenetic reprogramming of ISGs by mutant KRAS

**Figure 2.**
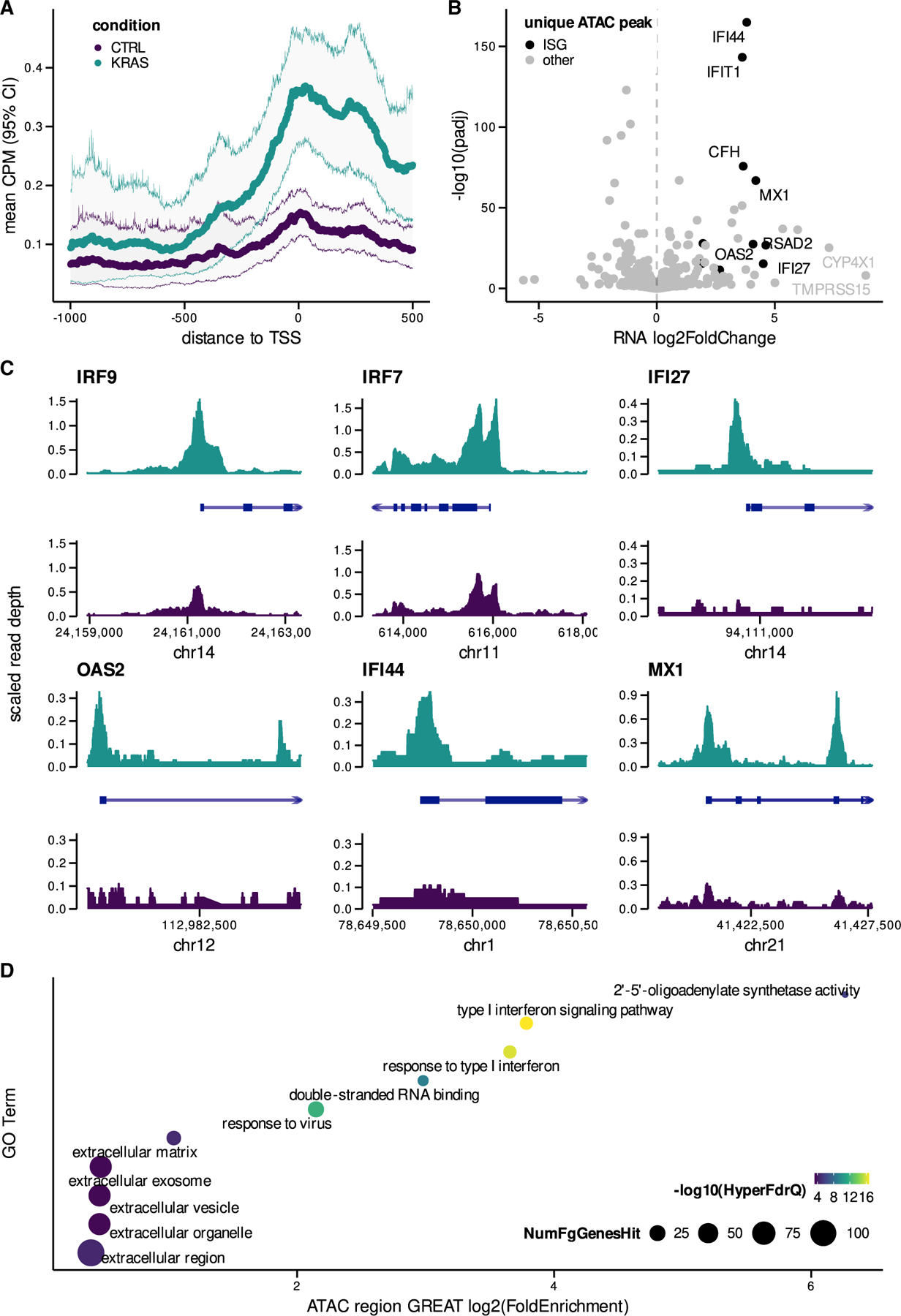
Mutant KRAS signaling mediates epigenomic reprogramming of ISGs (A) Mean ATAC-seq counts per million (CPM) (95% confidence interval [CI]) in promoter regions of upregulated ISGs (log2 fold change >1.5) in both mutant KRAS and control (CTRL) AALEs. (B) Differential expression of ISGs with unique peaks near TSS (only present in mutant KRAS or control AALEs). (C) ATAC-seq coverage in both mutant KRAS and CTRL AALEs for subset of ISGs with unique peaks detected near TSS. (D) Significant Gene Ontology (GO) term enrichment over unique peaks detected in mutant KRAS AALEs as determined by genomic regions enrichment of annotations tool (GREAT) analysis.

To elucidate potential mechanisms involved in inducing ISG signatures in mutant KRAS AALE cells, we performed the assay for transposase-accessible chromatin using sequencing (ATAC-seq) (Buenrostro et al., 2015). In mutant KRAS AALEs, open chromatin was significantly enriched at gene promoters for upregulated ISGs (Figure 2A). Open chromatin peaks were uniquely present in mutant KRAS AALEs when compared to control AALEs at 183 transcriptional start sites (TSSs), including 11 ISGs that were specifically and significantly upregulated by mutant KRAS signaling (Figure 2B). In addition, we observed strong enrichment of ATAC signal at the TSS of the significantly upregulated IRF9 gene, which forms the ISGF3 transcription factor (TF) complex with STAT1 and STAT2 (MacMicking, 2012), and also strong enrichment at the TSS of IRF7, IFI27, OAS2, IFI44, and MX1 (Figure 2C). In conjunction with the motif enrichment analysis (Figure 1D), these results show that oncogenic KRAS signaling induces the epigenetic activation of ISG TFs and their downstream ISG targets.

The genome-wide effects of mutant KRAS-mediated epigenomic reprogramming were further assessed with the Genome Regions Enrichment of Annotations Tool (GREAT) (McLean et al., 2010). GREAT analysis orthogonally confirmed the enrichment of accessible chromatin regions near ISGs and showed the enrichment of related molecular functions, including double-stranded RNA binding. Notably, the cellular components most enriched were extracellular in nature, including extracellular vesicle and extracellular exosome (Figure 2D).

### Mutant KRAS reprograms the extracellular transcriptome

**Figure 3.**
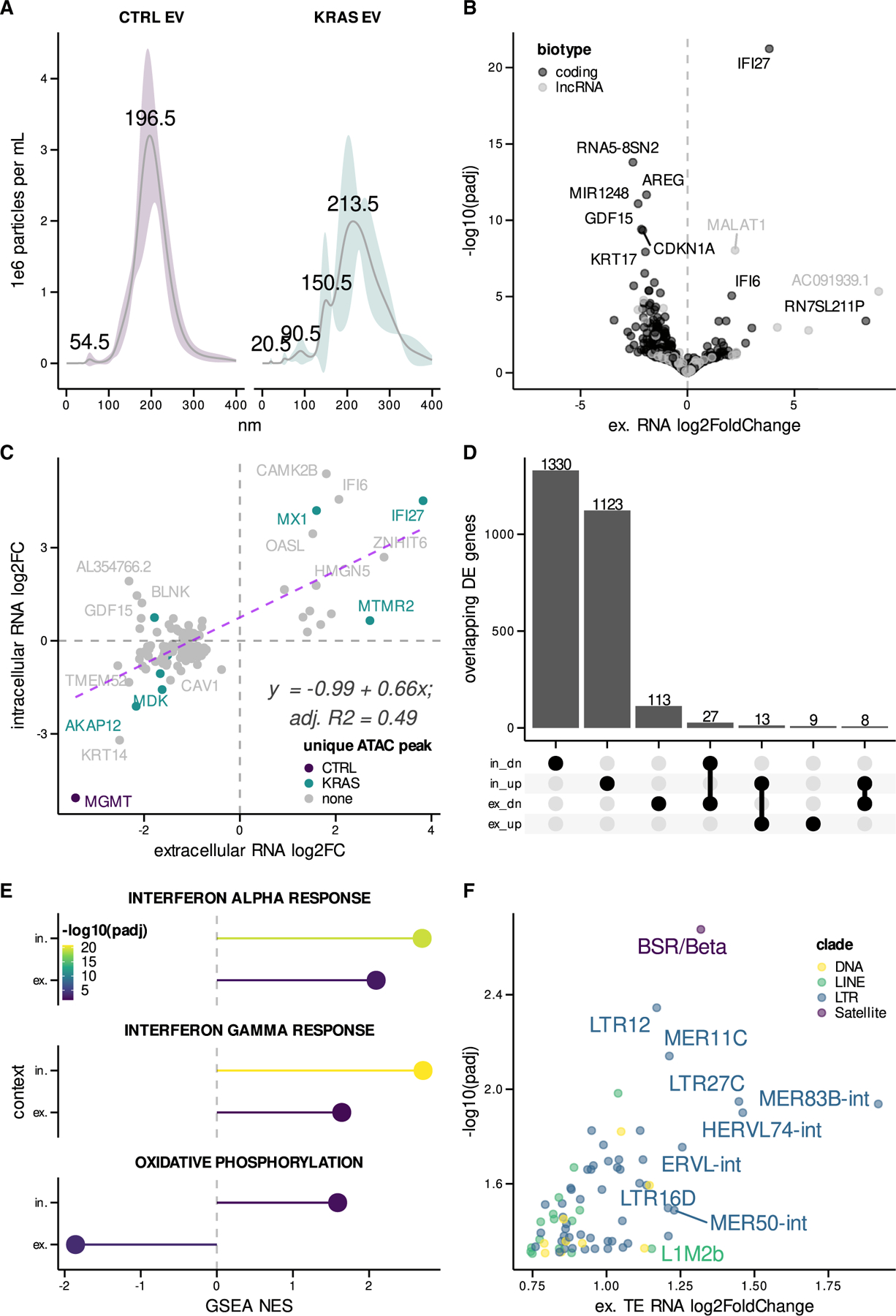
Mutant KRAS signaling induces secretion of TE RNAs and ISGs in EVs (A) Size distribution of extracellular vesicles (EV) isolated from control (CTRL) and mutant KRAS AALEs. (B) Volcano plot of differentially secreted GENCODE protein-coding RNAs and lncRNAs between mutant KRAS and CTRL AALE EVs. (C) Scatterplot comparing differentially expressed genes between intracellular and extracellular mutant KRAS AALE RNA-seq libraries; linear regression fit with formula and goodness of fit displayed. (D) Upset plot summarizing overlap of differentially expressed upregulated (up) and downregulated (dn) genes across in and ex contexts. (E) Significantly enriched gene sets detected in both in and ex contexts. (F) Differential secretion of TE RNAs in EVs from mutant KRAS AALEs when compared to control AALE EVs. Ex, extracellular; in, intracellular.

To test whether extracellular RNAs secreted from mutant KRAS cells also exhibit differential expression relevant to intracellular reprogramming events, we isolated extracellular vesicles from the culture media of control and mutant KRAS AALEs (Enderle et al., 2015; Liu et al., 2017). Extracellular vesicles isolated from mutant KRAS AALEs comprised different sized vesicles that were ~90, ~150, and ~213 nm in diameter, while vesicles from control AALE media were predominantly ~196 nm in size (Figure 3A).

RNA isolated and sequenced from these vesicles exhibited mutant KRAS-dependent differential expression of both protein-coding genes (n = 17 upregulated, n = 140 downregulated) and lncRNA (n = 5 upregulated, n = 8 downregulated) (Figures 3B and S2A). We also observed significant correlation between differentially expressed ISGs in our intracellular and extracellular RNA-seq datasets that largely agreed with intracellular epigenetic changes (IFI6, MX1, IFI27, and OASL) (Figures 3C and 3D). Furthermore, GSEA showed that IFN-*α* and -γ signatures were enriched in both intracellular and extracellular RNA (Figure 3E), indicating that extracellular RNAs reflect intracellular ISG changes due to mutant KRAS signaling.

To determine the effects of oncogenic KRAS on noncoding RNA secretion, we also characterized the TE RNAs that were preferentially packaged and released in extracellular vesicles. We found significant upregulation of predominantly long terminal repeat (LTR) RNAs such as LTR12, MER11C, and LTR27C, along with LINE, DNA, and Satellite repeat RNAs in mutant KRAS AALE extracellular vesicles (Figure 3F). Moreover, TE RNAs represented approximately 50% of the extracellular RNA released from mutant KRAS AALE cells, suggesting their preferential secretion in extracellular vesicles (Figures S2C and S2D).

### Regulation of TE RNAs by mutant KRAS

Given the prevalence of secreted TE RNAs, we investigated intracellular TE RNA dynamics in response to mutant KRAS signaling in AALE cells. Analogous to extracellular RNAs, LTR RNAs were among the most significantly upregulated TE RNAs in response to oncogenic KRAS signaling, including LTR12C RNAs (Figure S3A). In addition, LINE RNAs such as L1MEc and DNA element RNAs such as Tigger5 were also significantly enriched in mutant KRAS AALEs (Figure S3A). Furthermore, we examined TE RNAs in mutant KRAS HBEC cells, which similarly exhibited significant upregulation of TE RNAs in response to mutant KRAS when compared to control HBECs (Figure S3A). LTR12C RNAs were again the most significantly upregulated TE RNAs in mutant KRAS HBEC cells (Figure S3A), further validating our intracellular and extracellular RNA analyses in mutant KRAS AALE cells.

Based on the functions of KZNF genes in silencing TE RNAs in other contexts (Imbeault et al., 2017), we examined whether KZNFs could be involved in TE RNA regulation in both mutant KRAS AALE and HBEC cells. Given the broad downregulation of KZNFs in mutant KRAS AALEs (Figure 1A), we also analyzed KZNF expression in mutant KRAS HBECs, which similarly exhibited significant downregulation of KZNFs, many of which overlap with KZNFs downregulated in mutant KRAS AALEs (Figure S3B). To determine the potential relationship between our upregulated TE RNAs and our downregulated KZNFs, we looked for significantly enriched motifs in TE RNAs using a previously described KZNF-specific motif set (Barazandeh et al., 2018), which confirmed the presence of binding motifs for significantly downregulated KZNFs in the significantly upregulated TE RNAs (Figure S3C). We also used the KNZF binding scores generated from previous chromatin immunoprecipitation sequencing (ChIP-seq) experiments (Imbeault et al., 2017) to rank TE RNAs targeted by KZNFs, finding that many of the upregulated TEs were among the top 10–20 targets of downregulated KZNFs in mutant KRAS AALEs (Figure S3D), their extracellular vesicles (Figure S4A), and in mutant KRAS HBECs (Figure S4B). We then computed the average log2 fold change of downregulated ZNFs with putative binding sites within upregulated TE RNAs, which confirmed a negative association across all three contexts of mutant KRAS transcriptional profiling (Figure S3E). These analyses point to a coordinated, TE-KZNF axis that is dysregulated by mutant KRAS.

### KZNFs repress TE RNAs and ISGs activated by mutant KRAS

To explore the mechanistic relationship between KZNFs and TE RNA expression, we examined mutant KRAS A549 lung cancer cells that overexpress ZNF257 or ZNF682 (Ito et al., 2020), both of which we found to be significantly downregulated by mutant KRAS signaling in AALE cells and putative regulators of dysregulated TE families (Figures 1A and S3). Differential expression analysis of RNA-seq data indicated significant downregulation of ISGs OAS1 and IRF9 in mutant KRAS A549 cells overexpressing either ZNF257 or ZNF682 (Figure S5A), as well as significant downregulation of TE RNAs that were upregulated by mutant KRAS in AALE cells (Figure S5B). These findings directly connect mutant KRAS-regulated KZNFs with control of TE RNA and ISG expression.

### Epigenetic silencing of KZNFs regulated by mutant KRAS signaling

**Figure 4.**
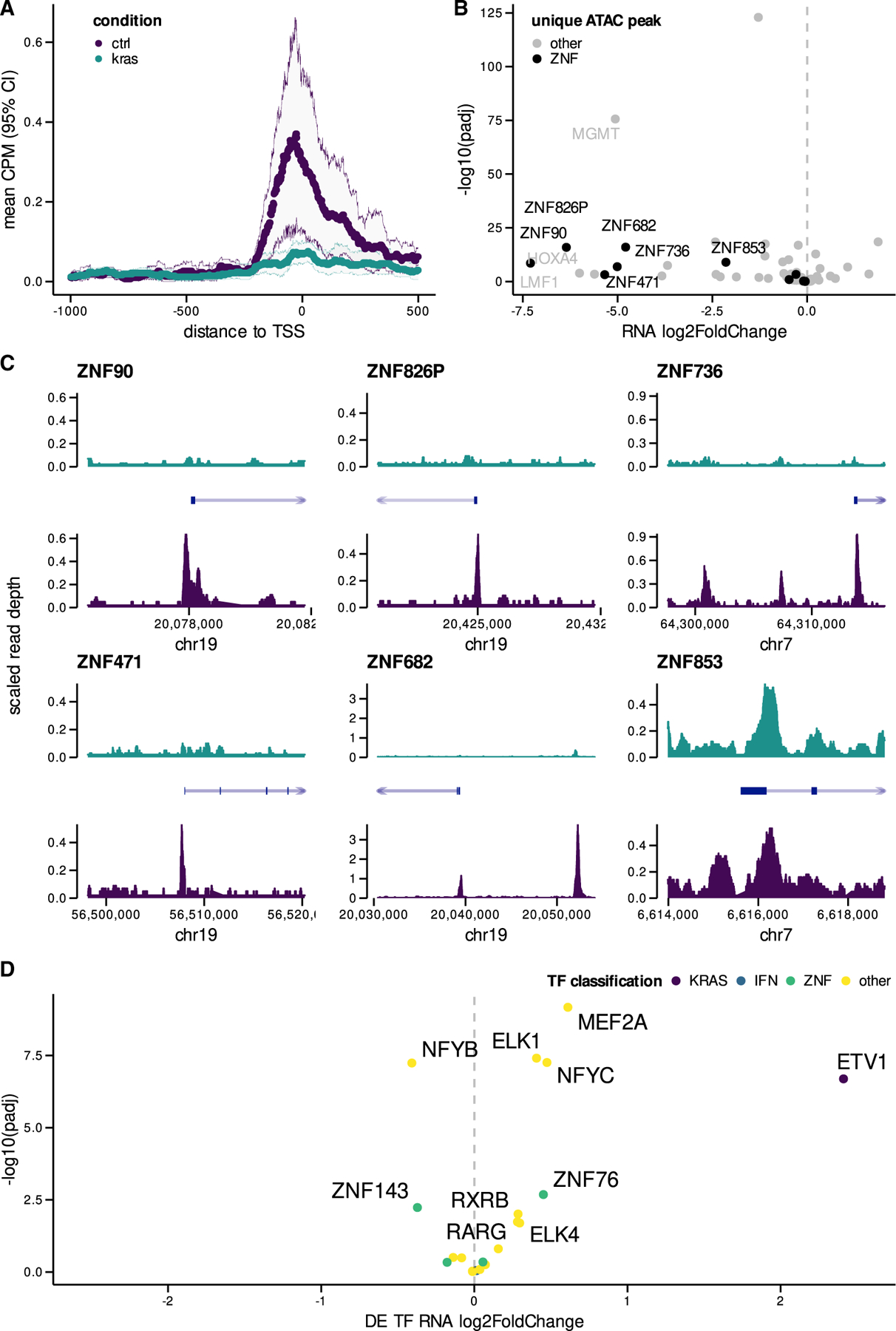
Mutant KRAS signaling epigenetically silences KZNFs *in vitro* (A) Mean ATAC-seq CPM (95% CI) in promoter regions of downregulated KZNFs (<–4.5 log2 fold change) in both mutant KRAS and control (CTRL) AALEs. (B) Differential expression of KZNFs with unique peaks near TSS (only present in mutant KRAS or control AALEs). (C) ATAC-seq coverage in both KRAS and CTRL AALEs for subset of KZNFs with unique peaks detected near TSS. (D) Volcano plots of differentially expressed TFs in mutant KRAS AALEs with significant TF motif enrichment in downregulated KZNF gene promoters. chr, chromosome.

To determine the extent to which mutant KRAS signaling epigenetically silences KZNF expression, we examined ATAC-seq data for all significantly downregulated KZNF loci. We found that mutant KRAS signaling substantially reduces chromatin accessibility at TSS regions (Figure 4A). When we examined genes with ‘‘unique’’ ATAC peaks that were only present in control AALEs but disappeared in mutant KRAS AALEs, we found that many of these genes were KZNFs that were significantly downregulated (Figure 4B). Six of these downregulated KZNFs, ZNF90, ZNF826P, ZNF736, ZNF471, ZNF682, and ZNF853, had peaks unique to control AALEs (Figure 4C). Downregulated KZNF TSS regions were enriched in motifs for ETS (ETV1) and ELK (ELK1) TFs (Figure 4D), known downstream effectors of the RAS signaling pathway (Simanshu et al., 2017).

### Downregulated KZNFs *in vivo* are associated with poor outcomes in lung cancer

**Figure 5.**
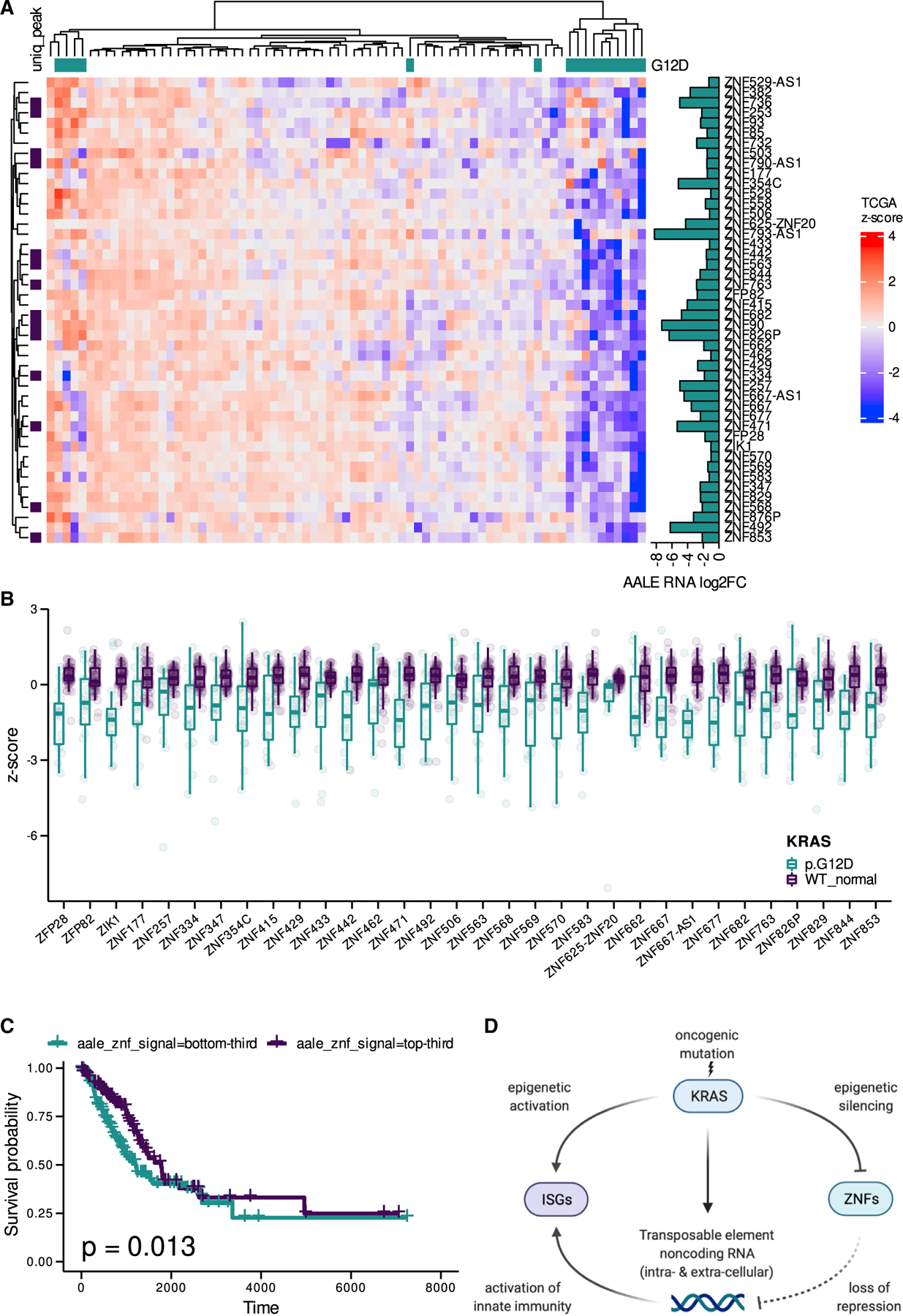
Broad downregulation of KZNFs in mutant KRAS LUAD *in vivo* (A) Hierarchical clustering of expression *Z* scores in TCGA LUAD RNA-seq data for KZNF genes downregulated in mutant KRAS AALEs; KZNFs with unique peaks in their promoter regions in control AALEs are labeled. (B) Distribution of *Z* scores for significantly downregulated KZNF genes (Wilcox) in TCGA LUAD RNA-seq data. (C) Kaplan-Meier survival curve for patients in the TCGA LUAD dataset stratified into thirds by expression levels of KZNFs downregulated in mutant KRAS AALEs. (D) Model of mutant KRAS-mediated regulation of TE RNAs and ISGs by KZNFs. Created with BioRender.com.

Finally, we explored the clinical significance of the mutant KRAS-induced KZNF silencing we identified in AALE and HBEC cells. Evaluation of KZNF expression in TCGA LUAD RNA-seq data revealed their significant downregulation in mutant KRAS(G12D) samples when compared to WT KRAS lung cancer or matched normal samples, respectively (Figures 5A and 5B). Furthermore, LUAD samples in the lowest third of KZNF expression demonstrated a significant decrease in overall survival probability (Figure 5C), highlighting the clinical impact of the mutant KRAS-mediated KZNF downregulation we found in AALE and HBEC cells.

## DISCUSSION

Collectively, our findings demonstrate the transcriptomic and epigenomic impact of oncogenic KRAS signaling on TE RNAs and ISGs. Our study suggests that KZNF repression by mutant KRAS signaling leads to de-repression of TE RNAs, triggering an intrinsic ISG response (Figure 5D). This model is supported by broad and significant downregulation of these same KNZFs in mutant KRAS-driven lung adenocarcinomas *in vivo*. Our conclusions are based on deeply sequencing and analyzing the intracellular and extracellular transcriptomes and epigenomes of mutant KRAS-transformed lung cells, building on previous work in which we discovered the coordinate regulation of noncoding RNAs and RAS signaling in the context of epigenomic reprogramming (Kim et al., 2015).

The molecular basis for the intrinsic ISG signature we observe in mutant KRAS AALE cells differs from TE RNA-induced IFN responses in cancer cells treated with DNA methyltransferase inhibitors (Chiappinelli et al., 2015; Roulois et al., 2015), as we instead find a prominent role for broad KZNF suppression during the early stages of mutant KRAS-driven cellular transformation. Our studies also suggest that oncogenic KRAS signaling is sufficient to induce at least a subset of the intrinsic ISG signatures that are observed across many cancers and cancer cells lines with ADAR dependencies (Gannon et al., 2018; Liu et al., 2019).

We also present further evidence for the utility of extracellular RNAs in detecting intracellular RNA changes in cancer cells (Reggiardo et al., 2022). Notably, we show the secretion of specific TE RNA and ISG signatures that are aberrantly upregulated in mutant KRAS lung cells. The enrichment of TE-derived noncoding RNAs in extracellular vesicles (Wang et al., 2021) released from mutant KRAS cells highlights their potential utility as RNA biomarkers for diagnosing RAS-driven cancers.

### Limitations of the study

Although we describe how mutant KRAS regulates the intracellular and extracellular transcriptomes in the context of lung cells, KRAS mutations are also prevalent in pancreatic and colorectal cancer cells. Additional studies using mutant KRAS pancreatic and colorectal cells, as well as additional analyses using corresponding TCGA RNA-seq data, would be needed to determine the broader physiological relevance of our findings across other cancer types driven by oncogenic RAS signaling. Moreover, while our results show that mutant KRAS is sufficient to activate a high ISG signature that is seen across many human cancers, additional experiments (e.g., CRISPR) that correct mutations in KRAS would reveal whether oncogenic RAS signaling is necessary for high ISG expression in tumor cells. Lastly, the biomarker potential of extracellular RNAs that are preferentially secreted from cancer cells with oncogenic RAS signaling would require validation using blood samples from patients with lung and other cancers having activating driver mutations in RAS pathway genes.

## STAR☆METHODS

### RESOURCE AVAILABILITY

#### Lead contact

Further information and requests for resources and reagents should be directed to and will be fulfilled by the lead contact, Daniel H. Kim (daniel.kim@ucsc.edu).

#### Materials availability

This study did not generate new unique reagents.

#### Data and code availability

- Bulk RNA-seq and ATAC-seq data have been deposited at GEO (GSE120566) and are publicly available as of the date of publication. Accession numbers are also listed in the key resources table. This paper also analyzes existing, publicly available data. These accession numbers for the datasets are listed in the key resources table.
- All original code has been deposited at Zenodo and is publicly available as of the date of publication. DOIs are listed in the key resources table.
- Any additional information required to reanalyze the data reported in this paper is available from the lead contact upon request.

### EXPERIMENTAL MODEL AND SUBJECT DETAILS

Immortalized lung epithelial cells (AALE cells; XX), derived at Dana-Farber and immortalized by SV40 large-T antigen (Lundberg et al., 2002) were obtained as a gift from the laboratory of Eric Collison (University of California, San Francisco). The AALE stable cell lines pBABE-mCherry Puro (control) (Lu et al., 2017) and pBABE-FLAG-KRAS(G12D) Zeo (mutant KRAS) were generated using retroviral transduction, followed by selection in puromycin or zeocin. Cells were cultured at 37°C and 5% CO_2_ in SABM Basal Medium (Lonza SABM basal medium, CC-3119) with supplements and growth factors (Lonza SAGM SingleQuots Kit Suppl. & Growth Factors, CC-4124).

HBEC3kt cell lines (HBEC cells; XX) were obtained as a gift from the laboratory of Harold Varmus (National Human Genome Research Institute and Weill Cornell Medicine). The HBEC stable cell lines pLenti6/V5-GW/lacZ (control) (Vikis et al., 2007) and pLenti-KRASV12 (mutant KRAS) were generated using lentiviral transduction, followed by selection in blasticidin. Lentiviral plasmids were obtained as a gift from the laboratory of John Minna (The University of Texas Southwestern Medical Center) (Sato et al., 2013; Vikis et al., 2007). Cells were cultured at 37°C and 5% CO_2_ in Keratinocyte Serum-Free Media (KSFM) with supplements (Invitrogen, #17005042).

### METHOD DETAILS

#### RNA-seq

For AALE cell lines, bulk RNA was isolated from cells using Quick-RNA MiniPrep kit (Zymogen) and RNA was quantified via NanoDrop-8000 Spectrophotometer. 1ug of total RNA was used as input for the TruSeq Stranded mRNA Sample Prep Kit (Illumina) according to manufacturer protocol. Library quality was determined through the High Sensitivity DNA Kit on a Bioanalyzer 2100 (Agilent Technologies). 6 multiplexed libraries, 3 biological replicates of each condition, were sequenced as HiSeq400 100PE runs.

For HBEC cell lines, cells grown in 10 cm plates (n = 3 per cell line) were washed twice in cold DPBS then collected in Tri-reagent for storage at –80°C until the bulk RNA was extracted using Direct-Zol RNA Miniprep Kit (Zymo Research). Concentrations of purified RNA in nuclease-free water were determined by Nanodrop-2000 Spectrophotometer and by Qubit RNA BR Assay (ThermoFisher Scientific). Quality RIN numbers ranging from 9.4–10 were determined by TapeStation 4150 RNA ScreenTape Analysis (Agilent Technologies) before sending RNA to UC Davis DNA Technologies and Expression Analysis Core Laboratory for poly-A strand specific library preparation to obtain 60 million paired end reads by NovaSeq S4 (PE150) sequencing.

#### ATAC-seq

100,000 AALE cells were collected and centrifuged at 500xg for 5 min at 4C. Pellets were washed with ice-cold PBS and centrifuged. Pellets were resuspended in ice-cold lysis buffer. Tagmentation reaction and purification were conducted according to manufacturer’s protocol (Active Motif). 2 Libraries, one from each condition, were sequenced on a NextSeq500 as 2 × 75 paired end reads.

#### Extracellular RNA-seq

The exoRNeasy serum/plasma maxi kit (Qiagen) was used to isolate extracellular vesicles, which were quantified using Nanoparticle Tracking Analysis (Malvern, UK). 30 mL of AALE cell culture supernatant was filtered to remove particles larger than 0.8 um. The filtrate was precipitated with kit buffer and filtered through a column to collect extracellular vesicles. These vesicles were then lysed with QIAzol® lysis reagent. Total RNA was isolated using the indicated phase separation method and used to make 6 libraries, 3 biological replicates for each condition, for RNA-seq using the Smart Seq HT low input mRNA library prep kit (Takara). Libraries were sequenced on an Illumina NextSeq500.

#### RNA-seq analysis

All *fastq* files were trimmed with *Trimmomatic 2 (0*.*38)* (Bolger et al., 2014) using the Illumina NextSeq PE adapters. The resulting trimmed files were assessed with *FastQC* (Brown et al., 2017) and then processed with the following analytical pipeline:

##### Salmon (1.3.0)

pseudoalignment of RNA-seq reads performed with *Salmon* (Patro et al., 2017) using the following arguments: –validateMappings –gcBias –seqBias –recoverOrphans –rangeFactorizationBins 4 using an index created from the *GENCODE* version 35 (Frankish et al., 2021) transcriptome fasta file using decoy sequences to enable selective alignment. An additional, TE-aware index was created in a similar fashion but supplemented with sequences generated from the UCSC Repeat Masker track.

##### DESeq2 (1.32.0)

*Salmon* output was imported into a DESeq object using *tximeta* (Love et al., 2020; Soneson et al., 2015) and differential expression analysis was performed with standard arguments (Love et al., 2014; Zhu et al., 2019). All results were filtered to have padj <0.05. In the case where R could only generate 0.00 for the padj values, they were reset to the lowest non-zero padj value in the dataset. Where count data was used, it was normalized across samples using DESeq.

#### Principal component analysis

PCA was performed in *R* using the function prcomp provided by the package *stats* (*4*.*1*.*1)*. Input gene abundance data was first variance stabilized using DESeq2 and then filtered for genes with 0 standard deviation across the samples.

#### Motif discovery and enrichment analysis

All motif-based analysis was performed in *R* using packages *memes (1*.*1*.*4)*, *universalmotif (1*.*10*.*2)*, *BSgenome*.*Hsapiens*.*UCSC*.*hg38 (1*.*4*.*3)*, *MotifDBGenomicRanges (1*.*44*.*0)* and *MotifDB* (1.34.0) (Bioconductor; Lawrence et al., 2013; Nystrom and McKay, 2021). Enriched motifs were identified by using the runAME() function provided by *memes* with a control set to ‘shuffle’ the input sequences unless otherwise noted in the text. Individual motif occurrences were identified with the runFimo() function provided by *memes*.

#### Zinc finger gene analysis

ChIP-exo data and supplementary information were extracted from supplementary data provided by Imbeault et al. (Imbeault et al., 2017). ZNF genes were cross referenced with *DESeq2* and bed file of Repeat Masked TE inserts from the UCSC Genome Browser to extract relevant differential expression data of ZNF proteins and Transposable Element transcripts using *R*. Promoter and motif analyses performed as described above.

Motif discovery was intersected with repeat-masked insertions and cross referenced with ChIP-exo target data to identify potential regulatory targets of differentially expressed KZNFs. KZNF targets were ranked by the score provided. Additional ZNF binding motifs were acquired from Barazandeh et al.’s website (Key resources table) and converted to a database compatible with MEME suite (Bailey et al., 2015; Barazandeh et al., 2018).

#### Gene set enrichment analysis

Differentially expressed genes were ranked by the shrunken log2FoldChange values generated by *DESeq2*. Gene sets were acquired using the *R* package *msigdbr (7*.*4*.*1)* (Dolgalev, 2022) and filtered to only contain gene sets with ‘Hallmark’ status.

The *R* package *fgsea (1*.*18*.*0)* (Korotkevich et al., 2016) was used to generate Gene Set Enrichment (Liberzon et al., 2011; Subramanian et al., 2005) estimates which were filtered to results with adjusted pvalues <0.05.

#### GREAT gene ontology analysis

The *R* package *rGREAT (1*.*24*.*0)* (Bioconductor) was used to process ATAC-seq identified peaks with GREAT and identify enriched GO terms. ATAC-seq peaks unique to either CTRL or KRAS contexts were used as input with the background set to the entire peak library comprised from both contexts.

#### TCGA ZNF analysis

TCGA-LUAD phenotype and normalized count data were downloaded from the UCSC Xena browser TOIL data repository (Key resources table) (Goldman et al., 2020). The files were combined and patients were grouped by their KRAS mutation status and identity. Heatmaps and associated hierarchical clustering were performed in *R* using the package *ComplexHeatmap* (2.8.0) (Gu et al., 2016). Survival analysis was performed using the *survival* R package (3.3) (Therneau, 2022).

#### ATAC-seq analysis

The nf-core ATAC-seq pipeline was used to process ATAC-seq reads to alignments with BWA, narrow peak calls with MACS2, and ultimately annotated peaks. Read count analysis was performed with the *R* package *bamsignals* (1.24.0) (Bioconductor) using the sorted bam files produced by the nf-core pipeline.

### QUANTIFICATION AND STATISTICAL ANALYSIS

All quantitative data for functional assays have been reported as means ± standard deviation. Statistical significance for these were calculated using a Wilcox-test (*R – wilcox*.*test()*) unless otherwise noted and p values <0.05 were considered significant. All statistical analyses were performed with R (version 4.1.1) running from the Rocker ‘Tidyverse’ Docker container (rocker/tidyverse:4.1.1). Linear regression was carried out with the lm() function.

### ADDITIONAL RESOURCES

UCSC Genome Browser tracks generated from ATAC-seq data: https://genome.ucsc.edu/s/rreggiar/aale%2DKRAS%2DG12%2Dtransformation.

## Supplementary Material

1

## Figures and Tables

**Table:** KEY RESOURCES TABLE

REAGENT or RESOURCE	SOURCE	IDENTIFIER

Critical commercial assays		

TruSeq Stranded mRNA Sample Prep Kit	Illumina	20020594
Bioanalyzer HS DNA	Agilent	5067–4626
Qubit RNA BR	ThermoFisher	Q32852
RNA ScreenTape	Agilent	5067–5576
Quick-RNA Miniprep	Zymogen	R1054
Direct-Zol RNA Miniprep	Zymogen	R2050
ATAC-seq Kit	Active Motif	53150
ExoRNeasy Serum/Plasma Maxi Kit	Qiagen	77064
Smart Seq HT mRNA Sample Prep Kit	Takara	634456

Deposited data		

AALE RNA-seq and ATAC-seq raw data	This paper	GEO: GSE120566
HBEC RNA-seq raw data	This paper	GEO: GSE120566
A549 ZNF overexpression data	Ito et al. (2020)	GEO: GSE78099
GENCODE v35	Frankish et al. (2021)	https://www.gencodegenes.org/human/release_35.html
TCGA counts data	UCSC Xena Browser	https://xenabrowser.net/datapages/?cohort=TCGA%20TARGET%20GTEx&addHub=https%3A%2F%2Fxena.treehouse.gi.ucsc.edu&removeHub=https%3A%2F%2Fxena.treehouse.gi.ucsc.edu%3A443
ZNF target database & scores	Imbeault et al. (2017)	https://static-content.springer.com/esm/art%3A10.1038%2Fnature21683/MediaObjects/41586_2017_BFnature21683_MOESM107_ESM.xlsx
Transposable element reference	UCSC Genome Browser	https://genome.ucsc.edu/cgi-bin/hgTables
KZNF binding motifs	Barazandeh et al. (2018)	http://kznfmotifs.ccbr.utoronto.ca/index.html

Experimental models: Cell lines		

Human lung airway epithelial cells (AALE)	Lundberg et al. (2002)	N/A
Human lung bronchial epithelial cells (HBEC3kt)	Harold Varmus lab	RRID:CVCL_X491

Recombinant DNA		

pBABE-FLAG-KRAS(G12D) Zeo	Addgene	RRID:Addgene_58902
pBABE-mCherry Puro	Lu et al. (2017)	RRID:Addgene_25896
pLenti6/V5-GW/lacZ	John D. Minna lab, Vikis et al. (2007), ThermoFisher	V49610
pLenti-KRASV12	John D. Minna lab, Vikis et al. (2007), Sato et al. (2013)	Backbone: V49610; Sequence: RRID:Addgene_12544

Software and algorithms		

FastQC (0.11.9)	https://www.bioinformatics.babraham.ac.uk/projects/fastqc/	https://github.com/s-andrews/FastQC/releases/tag/v0.11.9t
Original code	This paper	https://doi.org/10.5281/zenodo.6618294
Trimmomatic (0.39)	Bolger et al. (2014)	http://www.usadellab.org/cms/?page=trimmomatic
Salmon (1.3.0)	Patro et al. (2017)	https://salmon.readthedocs.io/en/latest/
nf-core/atacseq	https://github.com/nf-core/atacseq/tree/1.2.1	https://zenodo.org/record/3965985
*R (4.1.1)*	https://www.R-project.org/	R version 4.1.1 (2021–08-10) – ‘‘Kick Things’’
*R* – DESeq2	Love et al. (2014)	https://doi.org/10.18129/B9.bioc.DESeq2
*R* – apeglm	Zhu et al. (2019)	https://doi.org/10.18129/B9.bioc.apeglm
*R* – fgsea	Korotkevich et al. (2016)	https://doi.org/10.18129/B9.bioc.fgsea
*R* – tximeta	Love et al. (2020)	https://doi.org/10.18129/B9.bioc.tximeta
*R* – stats	R core team	https://www.R-project.org/
*R* – msigdbr	Dolgalev (2022)	https://cran.r-project.org/web/packages/msigdbr/index.html
*R* – GenomicRanges	Lawrence et al. (2013)	https://doi.org/10.18129/B9.bioc.GenomicRanges
*R* – BSgenome.Hsapiens.UCSC.hg38	Bioconductor	https://doi.org/10.18129/B9.bioc.BSgenome.Hsapiens.UCSC.hg38
*R* – survival	Therneau (2022)	https://cran.r-project.org/package=survival
*R* – rGREAT	Bioconductor	https://doi.org/10.18129/B9.bioc.rGREAT
*R* – MEMES	Nystrom and McKay, 2021	https://doi.org/10.18129/B9.bioc.memes
*R* – bamsignals	Bioconductor	https://doi.org/10.18129/B9.bioc.bamsignals
*R* – MotifDB	Bioconductor	https://doi.org/10.18129/B9.bioc.MotifDb
R – UniversalMotif	Bioconductor	https://doi.org/10.18129/B9.bioc.universalmotif
R – ComplexHeatmap	Gu et al. (2016)	https://doi.org/10.18129/B9.bioc.ComplexHeatmap

Other		

HTML code notebook, repo	This paper	https://github.com/rreggiar/aale-KRAS-G12-transformation

## Highlights

- Mutant KRAS signaling activates an intrinsic interferon-stimulated gene signature
- KRAB zinc-finger genes are silenced in mutant KRAS cells *in vitro* and *in vivo*
- Transposable element (TE) noncoding RNAs are upregulated by mutant KRAS signaling
- Mutant KRAS cells preferentially secrete TE noncoding RNA in extracellular vesicles
