# Supplementary material for: Mutant KRAS regulates transposable element RNA and innate immunity via KRAB zinc-finger genes
Source: Cell Rep. Author manuscript; Available in PMC 2022 Aug 12. (PMC9374308; doi:10.1016/j.celrep.2022.111104)
Supplement: 1 [file NIHMS1824946-supplement-1.pdf]

Cell Reports, Volume *40*

Supplemental information

Mutant KRAS regulates transposable element RNA and innate immunity via KRAB zinc-finger genes

Roman E. Reggiardo, Sreelakshmi Velandi Maroli, Haley Halasz, Mehmet Ozen, Eva Hrabeta-Robinson, Amit Behera, Vikas Peddu, David Carrillo, Erin LaMontagne, Lila Whitehead, Eejung Kim, Shivani Malik, Jason Fernandes, Georgi Marinov, Eric Collisson, Angela Brooks, Utkan Demirci, and Daniel H. Kim

SUPPLEMENTARY FIGURES

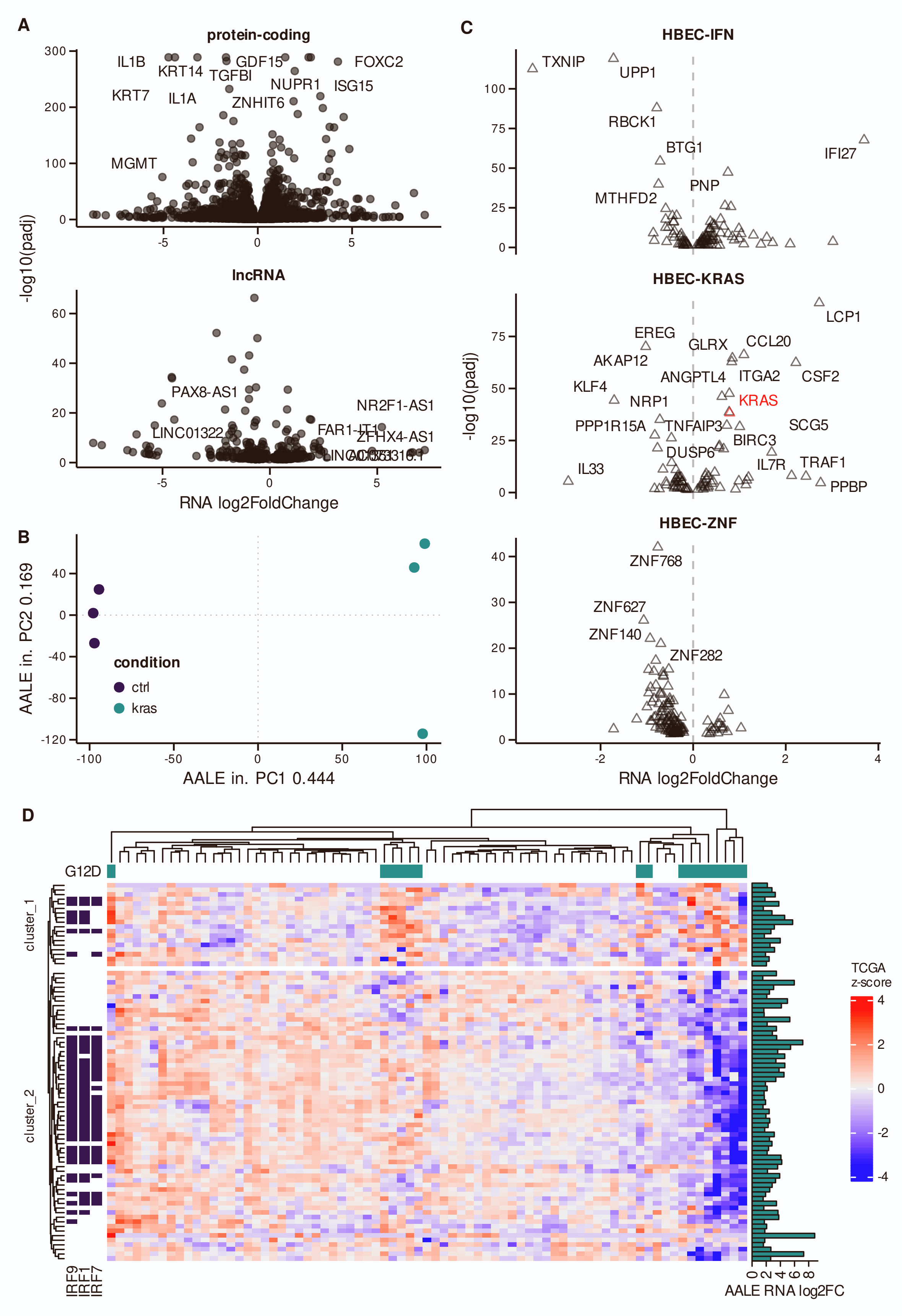

A
protein-coding
IL1B
KRT14
TGFBI
GDF15
FOXC2
NUPR1
ISG15
KRT7
IL1A
ZNHIT6
MGMT
-log10(padj)
lncRNA
PAX8-AS1
NR2F1-AS1
LINC01322
FAR1-IT1
ZFHX4-AS1
RNA log2FoldChange
B
AALE in. PC2 0.169
AALE in. PC1 0.444
condition
ctrl
kras
C
HBEC-IFN
TXNIP
UPP1
RBCK1
BTG1
IFI27
PNP
MTHFD2
HBEC-KRAS
LCP1
EREG
GLRX
CCL20
AKAP12
ANGPTL4
ITGA2
CSF2
KLF4
NRP1
KRAS
PPP1R15A
TNFAIP3
SCG5
BIRC3
DUSP6
IL7R
TRAF1
IL33
PPBP
HBEC-ZNF
ZNF768
ZNF627
ZNF140
ZNF282
RNA log2FoldChange
D
G12D
cluster_1
cluster_2
IRF9
IRF1
IRF7
TCGA
z-score
AALE RNA log2FC

Figure S1. Mutant KRAS signaling induces global transcriptional changes. Related to Figure 1.

A. Volcano plots of differentially expressed protein coding genes and lncRNAs in mutant KRAS AALEs. **B**. Principal component analysis (PCA) of control (ctrl) and mutant KRAS (kras) AALE RNA-seq libraries. Related to variance explained by each PC displayed with axis label. **C.** Volcano plots of differentially expressed protein coding genes and lncRNAs in mutant KRAS HBECs. **D.** Hierarchical clustering of expression z-scores in TCGA LUAD RNA-seq data for genes upregulated in mutant KRAS AALEs. Related to genes with IRF binding motifs in their promoter regions are labeled.

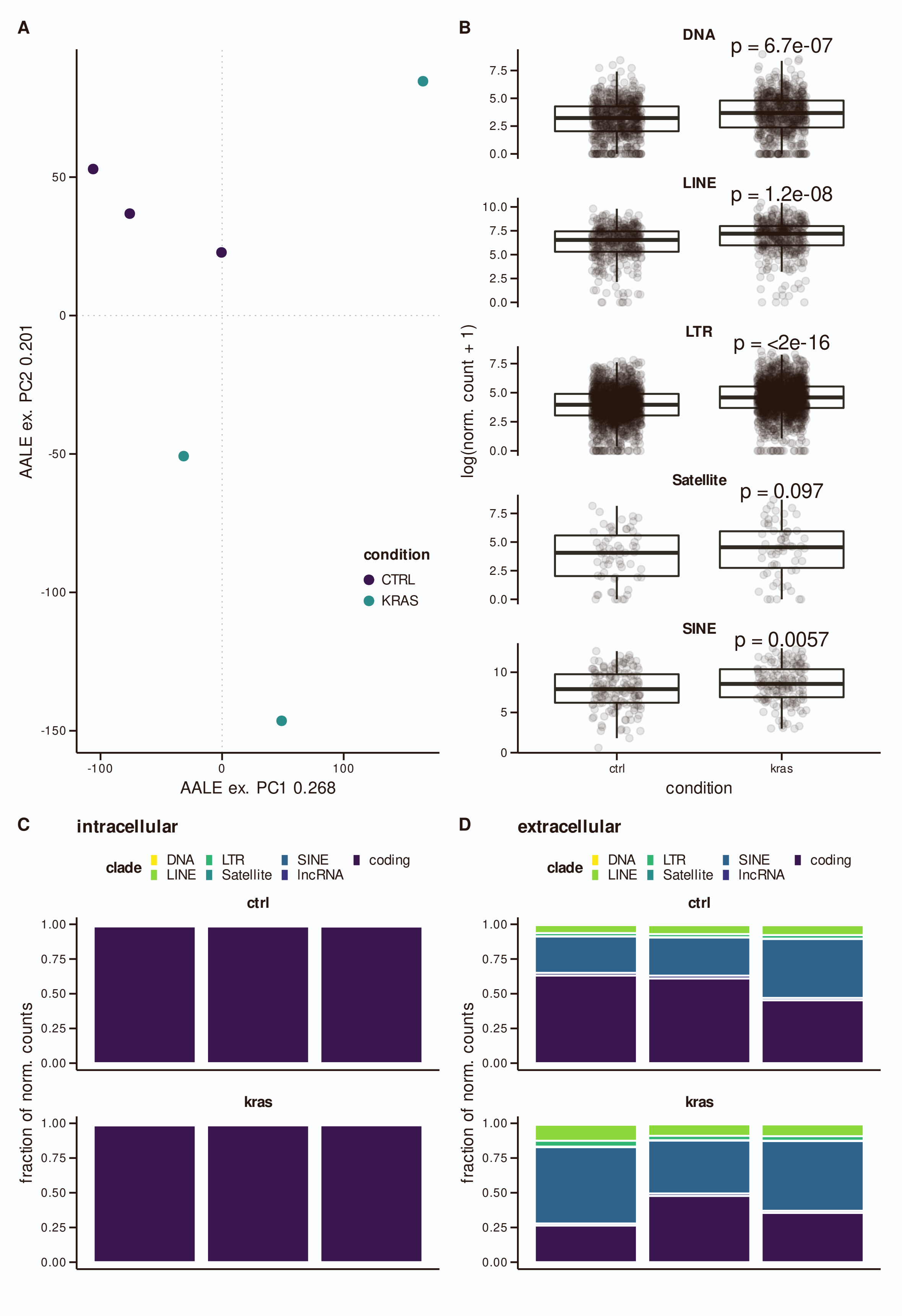
A
B
AALE ex. PC2 0.201
AALE ex. PC1 0.268
condition
CTRL
KRAS
DNA
p = 6.7e-07
LINE
p = 1.2e-08
LTR
p = <2e-16
Satellite
p = 0.097
SINE
p = 0.0057
log(norm. count + 1)
ctrl
kras
condition
C
intracellular
D
extracellular
clade
DNA
LINE
LTR
Satellite
SINE
lncRNA
coding
ctrl
kras
fraction of norm. counts

Figure S2. Mutant KRAS signaling significantly alters the RNA composition of secreted extracellular vesicles. Related to Figure 3.

A. Principal component analysis (PCA) of control (CTRL) and mutant KRAS (KRAS) AALE extracellular (ex) RNA-seq libraries. Related to variance explained by each PC displayed with axis label. **B**. Distribution of insertion-level abundance for TE clades in extracellular RNA-seq libraries (Wilcoxon). **C, D**. Distribution of counts assigned to GENCODE coding, lncRNA, and TE clades in intracellular and extracellular RNA-seq libraries.

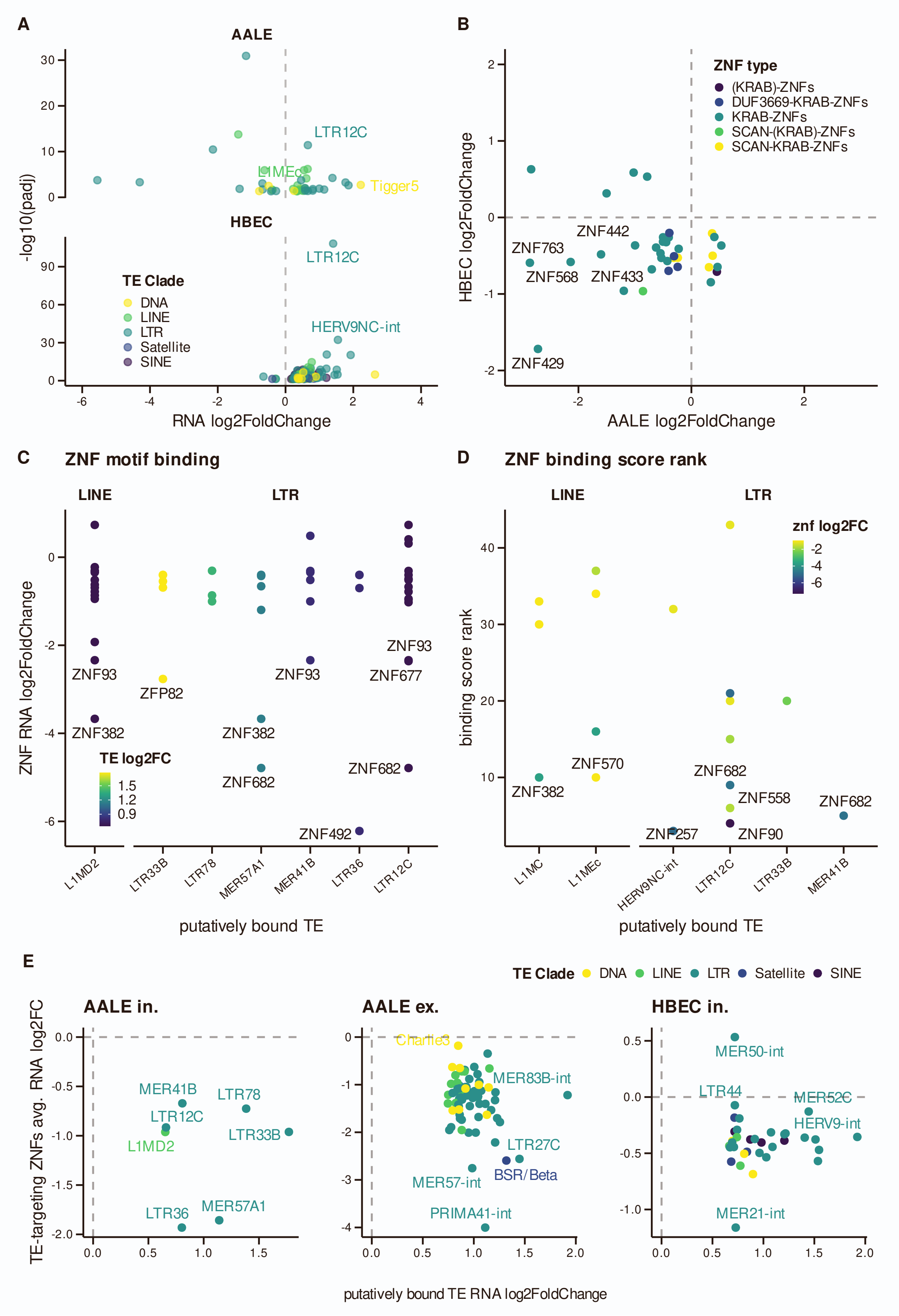

A
AALE
HBEC
LTR12C
L1MEc
Tigger5
HERV9NC-int
TE Clade
DNA
LINE
LTR
Satellite
SINE
-log10(padj)
RNA log2FoldChange
B
ZNF type
(KRAB)-ZNFs
DUF3669-KRAB-ZNFs
KRAB-ZNFs
SCAN-(KRAB)-ZNFs
SCAN-KRAB-ZNFs
ZNF442
ZNF763
ZNF568
ZNF433
ZNF429
HBEC log2FoldChange
AALE log2FoldChange
C
ZNF motif binding
LINE
LTR
ZNF93
ZFP82
ZNF382
ZNF682
ZNF677
ZNF492
TE log2FC
ZNF RNA log2FoldChange
L1MD2
LTR33B
LTR78
MER57A1
MER41B
LTR36
LTR12C
putatively bound TE
D
ZNF binding score rank
znf log2FC
ZNF382
ZNF570
ZNF682
ZNF558
ZNF257
ZNF90
binding score rank
L1MC
L1MEc
HERV9NC-int
putatively bound TE
E
TE Clade DNA LINE LTR Satellite SINE
AALE in.
AALE ex.
HBEC in.
MER41B
LTR78
LTR12C
L1MD2
LTR33B
LTR36
MER57A1
Charlie3
MER83B-int
LTR27C
MER57-int
BSR/Beta
PRIMA41-int
MER50-int
LTR44
MER52C
HERV9-int
MER21-int
TE-targeting ZNFs avg. RNA log2FC
putatively bound TE RNA log2FoldChange

Figure S3. TE RNAs activated by mutant KRAS are enriched for KZNF motifs. Related to Figures 3 & 4.

A. Volcano plots of differentially expressed TE RNAs in mutant KRAS AALEs and HBECs. **B**. Comparison of KZNF gene differential expression in mutant KRAS AALEs and HBECs. **C**. Differential expression of KZNFs with binding motifs in mutant KRAS-activated TE RNAs in AALEs. **D**. Ranking of KZNFs by binding score for mutant KRAS-activated TE RNAs in AALEs. **E**. Comparison of TE differential expression (x axis) to the average expression of ZNFs with putative binding sites within the TE based on motif library generated by Hughes *et al*.

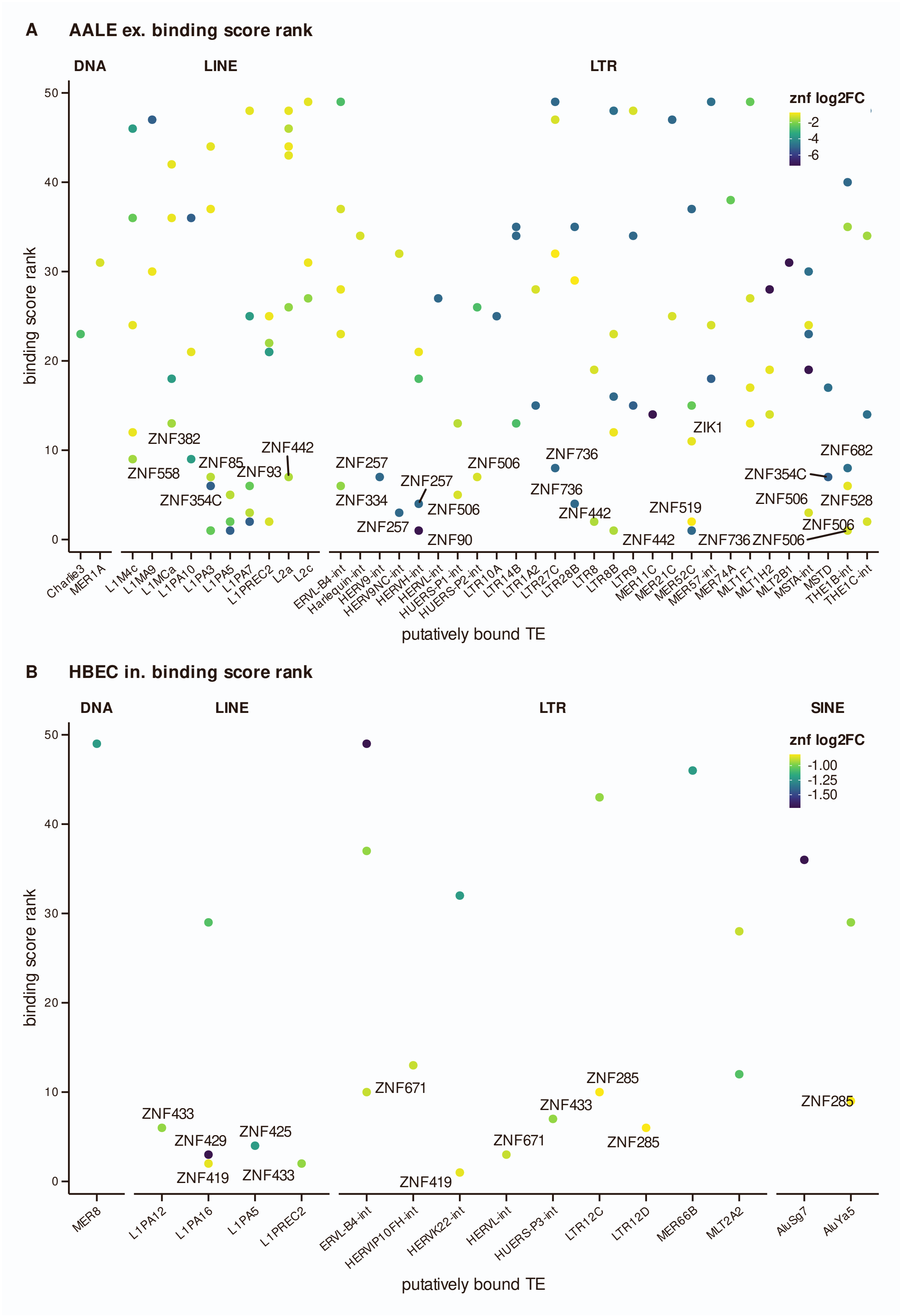
A
AALE ex. binding score rank
DNA
LINE
LTR
binding score rank
putatively bound TE
znf log2FC
ZNF382
ZNF558
ZNF85
ZNF93
ZNF442
ZNF354C
ZNF257
ZNF334
ZNF257
ZNF257
ZNF506
ZNF90
ZNF506
ZNF736
ZNF736
ZNF442
ZNF442
ZNF519
ZIK1
ZNF736
ZNF506
ZNF506
ZNF354C
ZNF682
ZNF506
ZNF528
B
HBEC in. binding score rank
DNA
LINE
LTR
SINE
binding score rank
putatively bound TE
znf log2FC
ZNF433
ZNF429
ZNF419
ZNF425
ZNF433
ZNF671
ZNF419
ZNF671
ZNF433
ZNF285
ZNF285
ZNF285

Figure S4. KZNF binding scores highlight potential regulation of differentially expressed TEs in mutant KRAS AALE EVs and HBEC cells. Related to Figures 3 & 4.

A,B. Ranking of KZNFs by binding score for each upregulated TE RNA in mutant KRAS extracellular (ex) AALE and intracellular (in) HBEC RNA-seq data.

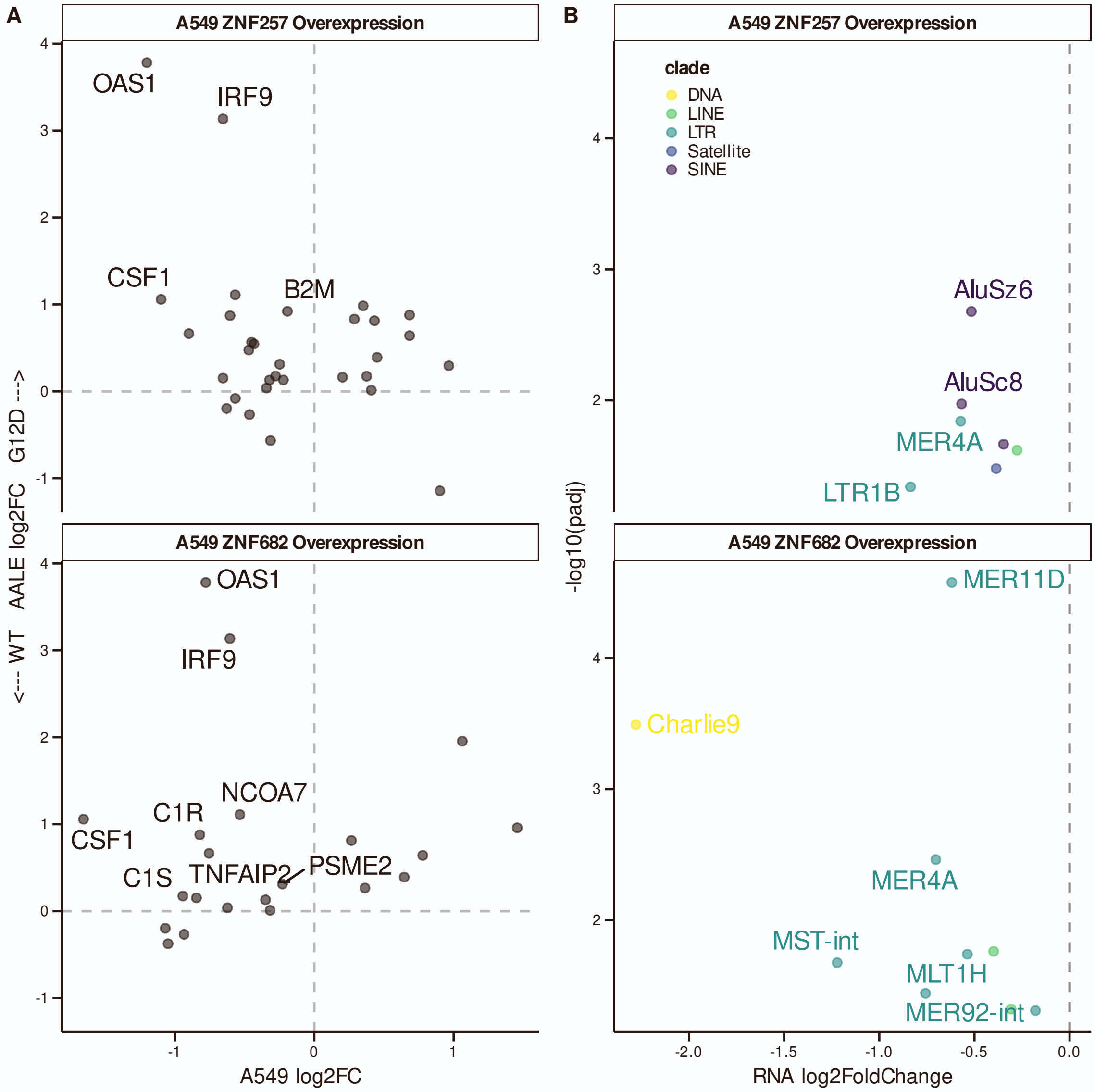
A
A549 ZNF257 Overexpression
OAS1
IRF9
CSF1
B2M
A549 ZNF682 Overexpression
OAS1
IRF9
NCOA7
C1R
CSF1
C1S
TNFAIP2
PSME2
<--- WT AALE log2FC G12D --->
A549 log2FC
B
A549 ZNF257 Overexpression
clade
DNA
LINE
LTR
Satellite
SINE
AluSz6
AluSc8
MER4A
LTR1B
A549 ZNF682 Overexpression
MER11D
Charlie9
MER4A
MST-int
MLT1H
MER92-int
-log10(padj)
RNA log2FoldChange

Figure S5. Mutant KRAS-regulated KZNFs repress ISGs and TE RNAs. Related to Figures 1, 2, 3 & 4.

A. Scatter plots of differentially expressed genes between mutant KRAS A549 lung cancer cells overexpressing ZNF257 or ZNF682 and mutant KRAS AALEs. **B**. Volcano plots of differentially expressed TE RNAs in mutant KRAS A549 lung cancer cells overexpressing ZNF257 or ZNF682.
